# Selective regimes and functional anatomy in the mustelid forelimb: Diversification toward specializations for climbing, digging, and swimming

**DOI:** 10.1002/ece3.3407

**Published:** 2017-09-20

**Authors:** Brandon M. Kilbourne

**Affiliations:** ^1^ Museum für Naturkunde Leibniz‐Institut für Evolutions‐ und Biodiversitätsforschung Berlin Germany

**Keywords:** adaptive diversification, limbs, locomotion, morphology, mustelidae, selective regimes

## Abstract

Anatomical traits associated with locomotion often exhibit specializations for ecological niche, suggesting that locomotor specializations may constitute selective regimes acting on limb skeletal traits. To test this, I sampled 42 species of Mustelidae, encompassing climbing, digging, and swimming specialists, and determined whether trait variation reflects locomotor specialization by performing a principal components analysis on 14 forelimb traits. In addition to Brownian motion models, three Ornstein–Uhlenbeck models of selective regimes were applied to PC scores describing trait variation among mustelids: one without a priori defined phenotypic optima, one with optima based upon locomotor habit, and one with a single phenotypic optimum. PC1, which explained 43.8% of trait variance, represented a trade‐off in long bone gracility and deltoid ridge length vs. long robustness and olecranon process length and distinguished between climbing specialists and remaining mustelids. PC2, which explained 17.4% of trait variance, primarily distinguished the sea otter from other mustelids. Best fitting trait diversification models are selective regimes differentiating between scansorial and nonscansorial mustelids (PC1) and selective regimes distinguishing the sea otter and steppe polecat from remaining mustelids (PC2). Phylogenetic half‐life values relative to branch lengths suggest that, in spite of a strong rate of adaptation, there is still the influence of past trait values. However, simulations of likelihood ratios suggest that the best fitting models are not fully adequate to explain morphological diversification within extant mustelids.

## 1 INTRODUCTION

Locomotion is fundamental to vertebrate biology, and anatomists have long noted that in many vertebrate taxa, particularly mammals (Dublin, 1903; Fish, Frappell, Baudinette, & MacFarlane, 2001; Hildebrand, 1985a, 1985b; Lull, 1904; Maynard Smith & Savage, 1956; Osburn, 1903; Polly, 2007; Samuels, Meachen, & Sakai, 2013; Samuels & Van Valkenburgh, 2008; Schutz & Guralnick, 2007; Shimer, 1903; Taylor, 1974, 1976, 1978; Van Valkenburgh, 1987), anatomical traits associated with locomotion are often specialized according to ecological niche. Notably, specializations in limb anatomy occur among the earliest mammaliform taxa, including specializations for climbing (Chen & Luo, 2013; Ji et al., 2002; Luo, Ji, Wible, & Yuan, 2003; Luo, Yuan, Meng, & Ji, 2011; Meng et al., 2015; Vázquez‐Molinero, Martin, Fischer, & Frey, 2001), digging (Luo, Meng, Liu, Zhang, & Neander, 2015; Luo & Wible, 2005; Martin, 2005), swimming (Ji, Luo, Yuan, & Tabrum, 2006; Martin, 2005), and gliding (Meng, Hu, Wang, Wang, & Li, 2006). Moreover, locomotor specializations in limb anatomy have been tied to lowered locomotor costs in a species' predominant habitat (Fish et al., 2001; Flaherty, Ben‐David, & Smith, 2010). The prevalence and early appearance of specialized limb anatomy in mammals and their close relatives, as well as their potential for lowered locomotor costs, suggest that morphological specializations in the locomotor system likely played a fundamental role in mammalian diversification.

Mustelidae are an ecologically diverse clade within Carnivora, having scansorial/climbing, fossorial/digging, and natatorial/swimming specialists, in addition to more generalized taxa (Holmes, 1980; Schutz & Guralnick, 2007). Moreover, Mustelidae are the most speciose family within Carnivora, with approximately 60 species, in addition to being a geologically young clade at roughly 16 mya (Sato et al., 2012). Interestingly, Mustelidae is now considered to exhibit widespread homoplasy in locomotor habit, with independent evolution of fossorial and natatorial specialists and terrestrial generalists (Koepfli et al., 2008; Sato et al., 2012). The species richness and ecological diversity within Mustelidae, in addition to its relatively young age, make Mustelidae an excellent candidate group with which to investigate limb functional morphology and trait diversification.

Among all the locomotor specializations exhibited by mustelids, the forelimbs play an important role, whether it is in digging (Hildebrand, 1985b; Moore, Budny, Russell, & Butcher, 2013; Rose, Moore, Russell, & Butcher, 2014), swimming (Fish, 1994; Williams, 1983a), climbing (Fabre et al., 2013b; Leach, 1977), or terrestrial locomotion (Gambaryan, 1974; Horner & Biknevicius, 2010; Williams, 1983b). Therefore, the anatomical traits of the forelimb in mustelids are likely strongly tied to the ecomorphological diversification of this clade. Here, I predict that fossorial, natatorial, scansorial, and generalized mustelids are distinguished by the functional anatomy of the forelimb skeleton, as it is reflected by bone length and diameter, and select muscle in‐levers. By fitting competing models of trait diversification, I will determine the most likely evolutionary process underlying forelimb skeleton diversity in my sample of mustelids. Given the forelimb's importance for the differing locomotor habits of mustelids, I predict that a model of adaptive diversification according to locomotor specialization is the most likely model of morphological diversification for the mustelid forelimb.

## 2 MATERIALS AND METHODS

### 2.1 Functional anatomy

**Figure 1:**
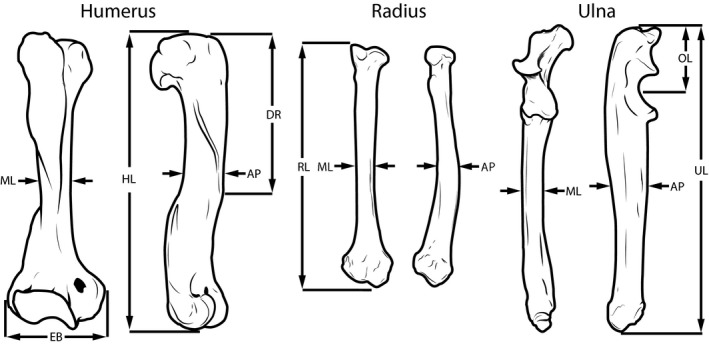
Linear dimensions of the humerus, radius, and ulna sampled for this study. HL, RL, UL, and OL, respectively, denote the lengths of the humerus, radius, ulna, and olecranon process. AP and ML, respectively, denote anteroposterior and mediolateral diameters at mid‐length. EB and DR, respectively, denote the epicondylar breadth and the length of the deltoid ridge

**Figure 2:**
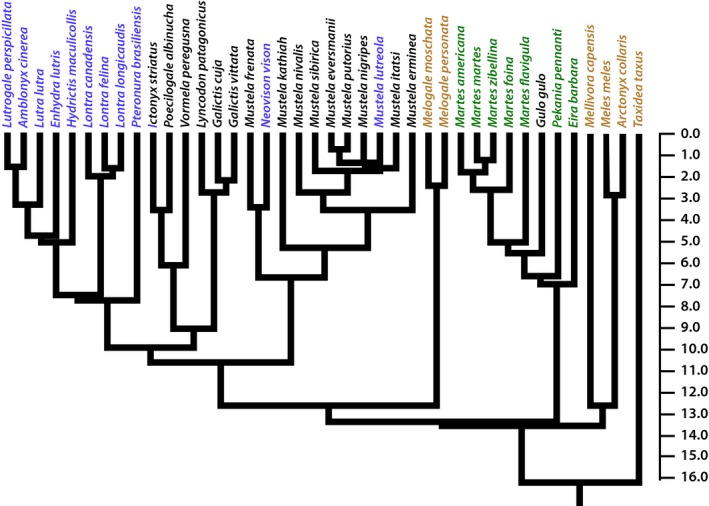
Phylogeny of extant Mustelidae sampled for this study, with branch lengths scaled to divergence times. Taxon names in blue denote taxa with a specialization for swimming (natatorial). Taxon names in brown denote taxa with a specialization for digging (fossorial). Taxon names in green denote taxa with a specialization for climbing (scansorial). Taxon names in black denote taxa with a generalized habit

Linear dimensions of the scapula, humerus, radius, ulna, and metacarpal III were collected from 42 species of mustelid (Table 1; Figure 1). These dimensions include bone lengths (with scapular length being measured along the scapular spine) and anteroposterior and mediolateral diameters at mid‐length along the humerus, radius, and ulna (Figure 2). Additionally, the lengths of the deltoid ridge of the humerus and the olecranon process of the ulna and the epicondylar breadth of the humerus were also measured. These traits are, respectively, in‐levers for the acromiodeltoid, the triceps brachii, and carpal flexors and extensors. The sample of Mustelidae represents roughly two‐thirds of mustelid species and broadly encompasses the locomotor specializations present in this clade (Table 1; Figure 1). Raw data are archived on Dryad Digital Respository (http://www.datadryad.org) with doi: DOI: 10.5061/dryad.87pg9.

**Table 1:** Mustelid taxa sampled for osteological traits

Species	Common name	*N*	Specialization	Body mass (g)
*Amblonyx cinerea*	Asian small‐clawed otter	4	Natatorial	3,990.4
*Arctonyx collaris*	Hog badger	1	Fossorial	6,356.0
*Eira barbara*	Tayra	5	Scansorial	3,910.0
*Enhydra lutris*	Sea otter	2	Natatorial	38,750.0
*Galictis cuja*	Lesser grison	4	Generalized	1,000.0
*Galictis vittata*	Greater grison	2	Generalized	3,200.0
*Gulo gulo*	Wolverine	3	Generalized	17,012.6
*Hydrictis maculicollis*	Spotted‐neck otter	4	Natatorial	4,000.0
*Ictonyx striatus*	Striped polecat	4	Fossorial	1,300.0
*Lontra canadensis*	N. American river otter	6	Natatorial	8,087.4
*Lontra felina*	Marine otter	2	Natatorial	4,500.0
*Lontra longicaudis*	Long‐tailed otter	3	Natatorial	6,555.0
*Lontra provocax*	Southern river otter	1	Natatorial	7,500.0
*Lutra lutra*	European river otter	2	Natatorial	8,785.1
*Lutrogale perspicillata*	Smooth‐coated otter	3	Natatorial	9,966.7
*Lyncodon patagonicus*	Patagonian weasel	1	Generalized	225.0
*Martes americana*	American marten	6	Scansorial	1,250.0
*Martes flavigula*	Yellow‐throated marten	2	Scansorial	2,500.0
*Martes foina*	Beech marten	4	Scansorial	1,540.8
*Martes martes*	Pine marten	2	Scansorial	1,300.0
*Martes zibellina*	Sable	2	Scansorial	1,130.0
*Meles meles*	European badger	3	Fossorial	13,000.0
*Mellivora capensis*	Honey badger / Ratel	2	Fossorial	8,000
*Melogale moschata*	Chinese ferret‐badger	3	Fossorial	938.5
*Melogale orientalis*	Javan ferret‐badger	2	Fossorial	2,000.0
*Melogale personata*	Burmese ferret‐badger	2	Fossorial	1,702.5
*Mustela erminea*	Ermine / Stoat	3	Generalized	168.8
*Mustela eversmanii*	Steppe polecat	1	Generalized	1,350.0
*Mustela frenata*	Long‐tailed weasel	6	Generalized	147.0
*Mustela itatsi*	Japanese weasel	2	Generalized	400.0
*Mustela kathiah*	Yellow‐bellied weasel	1	Generalized	208.08
*Mustela lutreola*	European mink	2	Natatorial	440.0
*Mustela nigripes*	Black‐footed ferret	4	Generalized	850.0
*Mustela nivalis*	Least weasel	2	Generalized	103.9
*Mustela putorius*	European polecat	4	Generalized	730.8
*Mustela sibirica*	Siberian weasel	1	Generalized	405.0
*Neovison vison*	N. American mink	6	Natatorial	945.0
*Pekania pennanti*	Fisher	6	Scansorial	4,000.0
*Poecilogale albinucha*	African striped weasel	4	Fossorial	340.0
*Pteronura brasiliensis*	Giant otter	3	Natatorial	23,999.9
*Taxidea taxus*	N. American badger	6	Fossorial	7,107.6
*Vormela peregusna*	Marbled polecat	1	Generalized	450.4

Note that *L. provocax* and *M. orientalis* were not included in trait diversification models, as these taxa were not included in previously published, fully resolved phylogenies. Body mass values are from Smith et al. (2003) and are provided for size comparison.

To discern differences in functional anatomy among differing locomotor specialists in Mustelidae, I performed a standard principal components analysis on mean values of each trait for each species. However, as body size strongly influences variation in functional anatomical traits (e.g., Fabre, Cornette, Peigné, & Goswami, 2013a), I first sought to minimize the influence of size. To this end, I performed ordinary least squares bivariate regressions between each of the 14 traits and the geometric mean calculated from these 14 traits. Prior to performing regressions, I log‐transformed both forelimb trait and geometric mean values. From the regressions, I then calculated residuals for each species along the *y* axis to obtain forelimb trait values with a minimized influence of size for use in the standard principal components analysis. To determine which of the *n* principal components were significant, I used the broken stick model (Borcard et al., 2011; Legendre & Legendre, 1998).

Principal components analyses and regressions were performed in R vers. 3.3.1 (R Core Team, 2016), and residuals from ordinary least squares regressions were calculated using the package smatr (Warton, Duursma, Falster, & Taskinen, 2012). Geometric means were calculated using the package pysch (Revelle, 2017).

### 2.2 Trait diversification

To determine the most likely model of forelimb trait diversification, competing models of Brownian motion and adaptive diversification were fit to the data with the best model being chosen by Akaike information criterion for small samples (AICc), Akaike weights, and parameter inspection (Cooper, Thomas, Venditti, Meade, & Freckleton, 2016) using the R package qpcR (Speiss, 2014). When fitting suites of traits to models of adaptive diversification, a decision has to be made to use a multivariate model (Clavel, Escarguel, & Merceron, 2015) or to use a univariate model fitting PC scores representing suites of traits (Collar, O'Meara, Wainwright, & Near, 2009; Friedman, Price, Hoey, & Wainwright, 2016). While arguably analytically better, using multivariate models for a large number of traits can lead to parameter estimates greatly exceeding the number of observations in the dataset, reduced analytical power, and difficulty in estimating model parameters.

To model adaptive diversification, I fitted PC scores to Ornstein–Uhlenbeck (OU) models. An OU model fits trait data to sets of phenotypic optima acting along the branches of a phylogeny; these optima constitute selective regimes (Beaulieu, Jhwueng, Boettiger, & O'Meara, 2012; Butler & King, 2004; Hansen, 1997; Uyeda & Harmon, 2014). Typically, the number and distribution of these optima across the branches of a phylogeny are defined a priori in OU models. However, OU models can also be fitted without a priori specifications of the number and locations of phenotypic optima on the phylogeny (i.e., an unconstrained model). The use of OU models with and without selective regimes defined a priori allows for not only testing which set of proposed selective regimes fits best but also whether the best fitting of proposed selective regimes is necessarily the best descriptor of the observed data. When fitting an unconstrained model, a posterior probability is generated for each proposed shift in selective regime. A shift in regime with a posterior probability greater than or equal to 0.20 was taken as statistically significant.

**Figure 3:**
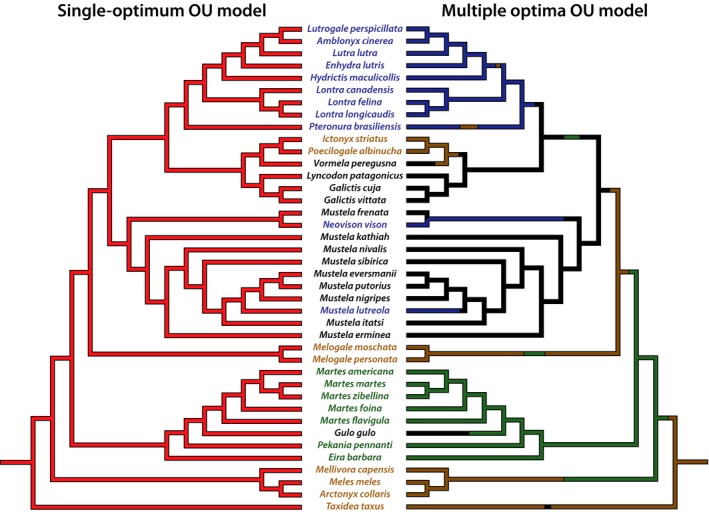
A priori defined Ornstein–Uhlenbeck (OU) and Brownian motion (BM) models of trait diversification. In the single‐optimum OU model, a single phenotypic optimum acts across all the branches of the phylogeny. The four optima of the multi‐optima OU model are based upon fossorial, natatorial, scansorial, and generalized locomotor habits (respectively represented by brown, blue, green, and black branches), and for this model, uncertainty in ancestral states of locomotor habits was incorporated with stochastic character mapping (see Section 2). The optima of the single‐optimum and multi‐optima OU models were also used as a basis for single‐rate and multirate Brownian motion models, with the latter also employing stochastic character mapping. Colors of taxon names denote the locomotor habits of sampled taxa as a reference

Of the a priori defined selective regimes, I proposed models with one and four phenotypic optima (Figure 3). The one optimum model corresponds to one phenotypic optimum that acts across all sampled branches of Mustelidae. The four optima model proposes a phenotypic optimum for each locomotor specialization (generalized, scansorial, fossorial, and natatorial mustelids). Models encompassing differing rates of Brownian motion can also lead to increased morphological disparity (O'Meara, Ané, Sanderson, & Wainwright, 2006). In light of this, I also fit a single‐rate Brownian motion model, and a four‐rate Brownian motion model. The four distinct rates corresponded to the four locomotor habits within Mustelidae.

For a priori defined selective regimes, although the locomotor specializations is only known for the phylogeny's terminal taxa, the distribution of specializations among internal branches is unknown. To incorporate uncertainty in the assigning of phenotypic optima to the tree's internal branches, I used stochastic character mapping. In stochastic character mapping, phenotypic optima are stochastically mapped onto the internal branches of the tree, then multi‐optima OU and multirate Brownian motion models are fitted, and model parameters are calculated. This random mapping of optima, modeling fitting, and the calculation of model parameters is performed over a total of 500 iterations, and the mean for each model parameter is calculated across the 500 iterations. Stochastic character mappings were performed with the R package phytools (Revell, 2012). To compare fits of unconstrained and a priori defined models, selective regimes generated in bayou were converted to OUwie format using bayou (Uyeda & Harmon, 2014).

Unconstrained OU models were fit using the R package bayou (Uyeda & Harmon, 2014), which uses a Bayesian Monte Carlo Markov Chain approach. Models fit by bayou were generated from 1,500,000 runs. All a priori defined OU and BM models were fit using the R package OUwie (Beaulieu et al., 2012). I also fit early burst and white noise models using the R package geiger (Harmon, Weir, Brock, Glor, & Challenger, 2008)—models of a decelerating rate of evolution and no phylogenetic signal, respectively. Parameter estimates for OU models are provided in Tables S1–S6.

#### 2.2.1 Null model comparison

To further test of the adequacy of model fit, I compared the best fitting model to a single‐rate Brownian motion model of trait diversification, which can be taken as a null model of trait evolution. Using simulations to compare the best fitting model to a null model can reveal the adequacy of the sampled phylogeny for model fitting and the adequacy of overall model fit, as the number of taxa and height of a tree can impair the fit of a model (Boettiger, Coop, & Ralph, 2012; Cooper et al., 2016). To do this, I followed the methodology of Boettiger et al. (2012) on the recommendation of Cooper et al. (2016). Data were simulated as evolving under the best fitting and null models. Then to each set of simulated data, the best fitting and null models were fit and the likelihood ratio was calculated from the likelihood scores of these two fits to a given simulated dataset. Thus, a likelihood ratio could be obtained for both sets of data simulated separately under null and best‐fit models. This process was repeated over 10,000 iterations, and two 95% confidence interval were generated—one for likelihood ratios generated under data simulated under the null model and another for data simulated under the best‐fit model. The likelihood ratio calculated from the empirical data was then used as a critical value, and its exclusion from either confidence interval reveals the adequacy of a phylogeny for fitting models of trait diversification (Boettiger et al., 2012; Cooper et al., 2016).

#### 2.2.2 Phylogeny

A phylogeny of sampled taxa was created by primarily using the phylogeny of Sato et al. (2012), with additional taxa added from Koepfli et al. (2008) (Figure 2). These two phylogenies had agreeing topologies, and differences in divergence times between these two phylogenies are largely minor. To add missing taxa in Sato et al. (2012) from Koepfli et al. (2008), added branches leading to missing taxa were scaled to the divergence time provided by Sato and colleagues for the most recent branch shared by both phylogenies that was ancestral to the added taxon. However, note that two taxa, *Lontra provocax* and *Melogale orientalis*, were omitted from the phylogeny, as neither of these taxa was included in either published phylogeny.

## 3 RESULTS

### 3.1 Locomotor specialization and morphology disparity

PC‐1 explains 43.8% of the total variance, whereas PC2 explained 17.4%. When using the broken stick model, only the variances of these two PCs exceed the expected variances (23.2% for PC1 and 16.1% for PC2). Thus, PCs 1 and 2 were taken as the only two significant PC axes.

**Figure 4:**
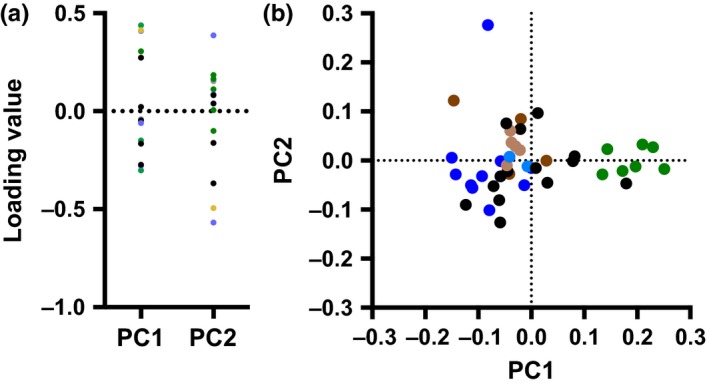
Phenotypic variability of the mustelid forelimb skeleton as modeled by PC1 and PC2, highlighting mustelid locomotor habits. The distribution of loadings along PC1 and PC2 are provided in a. Loadings highlighted in green, yellow, and periwinkle represent traits of the humerus, ulna, and radius, respectively. Loadings highlighted in gray and black represent traits of the scapula and metacarpal III, respectively. A plot of PC1 scores vs. PC2 scores is provided in b. Green points in b represent scansorial taxa, and blue points represent natatorial taxa. Light blue points represent minks, whereas dark blue points represent otters. Brown points represent fossorial taxa, with dark brown points representing fossorial taxa stemming from early divergences in Mustelidae (*Arctonyx*,* Meles*,* Mellivora*, and *Taxidea*) and light brown points representing fossorial taxa stemming from later divergences in Mustelidae (*Ictonyx*,* Melogale*, and *Poecilogale*). Black points represent generalized taxa

As PC1's loadings span a value of 0.0, this axis represents a trade‐off in forelimb morphology. At one extreme of the loadings for PC1 are the lengths of metacarpal III, the radius, humerus, ulna, and the deltoid ridge, whereas at the other extreme are the length of the olecranon process and the anteroposterior diameters of the radius, humerus, and ulna (Table 1; Figure 4). PC2's loadings represent a trade‐off between a larger radial mediolateral diameter and larger humeral anteroposterior and mediolateral diameters on one extreme and a longer third metacarpal and larger radial anteroposterior diameter on the other. It is worth noting that the magnitudes of loadings for humeral, radial, and ulnar lengths along PC2 are of a low magnitude, indicating these traits are not strongly reflected along this axis.

Plotting axes PC1 vs. PC2 (Figure 4) reveals that scansorial mustelids are morphologically disparate from remaining mustelids along PC1, having more elongate and thinner long bones, a longer deltoid ridge, and shorter olecranon process relative to other mustelids. However, the more generalized wolverine (*Gulo gulo*) also clusters with scansorial mustelids. Natatorial and fossorial mustelids occupy two largely distinct regions of phenotypic space due to their positions along both PCs 1 and 2 (Figure 4). Natatorial mustelids tend to have a relatively longer third metacarpal and a radius with a wide anteroposterior diameter, whereas fossorial mustelids tend to have a relatively more robust humerus and a radius with a wide mediolateral diameter. The position of generalized mustelids appears to be owed to both PC1 and PC2, and generalized mustelids span the region of phenotypic space occupied by both natatorial and fossorial mustelids.

### 3.2 Selective regimes and locomotor specialization

**Figure 5:**
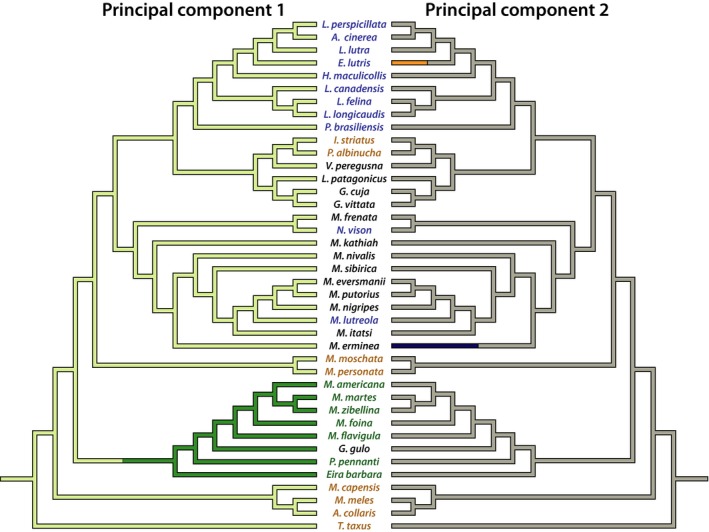
Unconstrained Ornstein–Uhlenbeck models for PC1 and PC2. Changes in color along branches indicate a shift in phenotypic optimum. Selective regimes were generated by the R package bayou (Uyeda & Harmon, 2014). Colors of taxon names denote the locomotor habits of sampled taxa as a reference: fossorial (brown), natatorial (blue), scansorial (green), and generalized (black)

Fitting an unconstrained OU model to PC axes yielded sets of selective regimes with one (PC1) and two (PC2) shifts in regime (Figure 5). In the unconstrained OU model for PC1, the shift in phenotypic optima occurs halfway along the branch leading to Guloninae (i.e., *Martes*,* Gulo*, and *Pekania*) with a posterior probability of 0.73. In the unconstrained OU model for PC2, shifts in phenotypic optima occur at roughly 40% of the length of branches leading to the sea otter (*Enhydra lutris*; posterior probability = 0.97) and the steppe polecat (*Mustela eversmanii*; posterior probability = 0.25). Notably, these two taxa possess the most extreme scores along PC2.

Comparing models reveals that the unconstrained OU model is the besting fitting model for both PCs 1 and 2, with the unconstrained model for PC1 having a 55.1% and the unconstrained model for PC2 having a 99.9% probability of being the best fitting model for their PC axes (Table 3). For the OU models best fitting PC1 and PC2, α = 0.26 and α = 0.11, respectively. These values of α, respectively, correspond to phylogenetic half‐lives of 2.65 and 6.25.

The likelihood ratio (19.7) for PC1's best fitting model vs. the null model (single‐rate Brownian motion) lies outside the 95% confidence intervals for likelihood ratios generated from both models. The likelihood ratio confidence interval from data simulated under the unconstrained OU model is (0.75, 2.55), whereas the confidence interval from data simulated under the null model is (0.21, 1.20). PC2's likelihood ratio (41.0) also lies outside of the confidence interval generated from its best fitting model (11.68, 19.44), as well as the confidence interval generated from the null model (0.05, 2.77). Thus for both PCs 1 and 2, the phylogeny for Mustelidae may have too few taxa to accurately fit OU models to PC axes.

## 4 DISCUSSION

### 4.1 Locomotor specialization and morphology disparity

**Figure 6:**
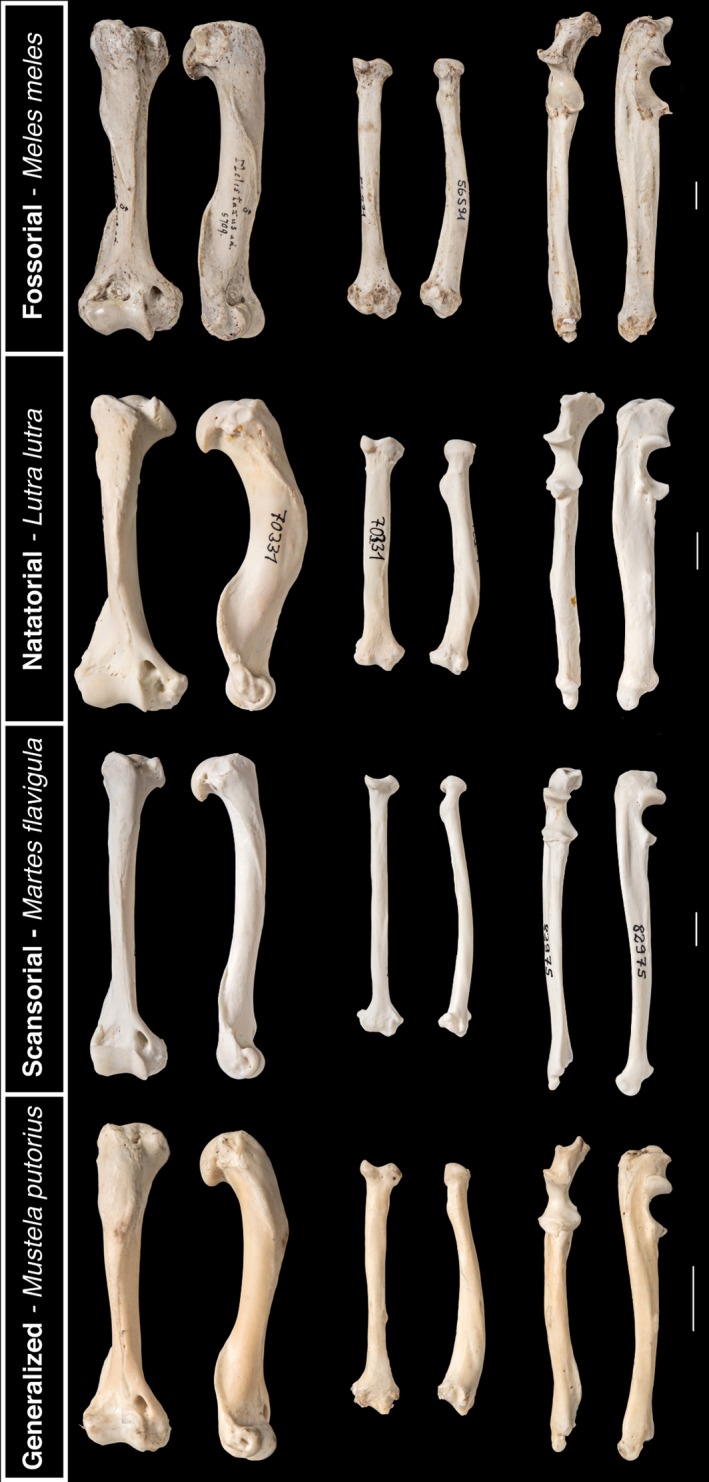
Examples of the long bone morphologies of fossorial, natatorial, scansorial, and generalized mustelids, with one example species for each locomotor specialization. Scale bars represent 1 cm

Scansorial, fossorial, and natatorial mustelids occupy largely distinct regions of phenotypic space (Figure 4). This distribution is largely attributable to both PCs 1 and 2, which cover roughly 61% of the variance in the data. The highest loadings along PC1 are for the length of the deltoid ridge and the lengths of the long bones and metacarpal III, whereas the lowest loadings are for the anteroposterior diameters of the long bones and olecranon process length (Table 2). These traits are key to distinguishing scansorial from nonscansorial mustelids (Figure 4). Compared to mustelids of other locomotor specialization, scansors possess a relatively longer deltoid ridge, more gracile long bones, and a shorter olecranon process (Figure 6). Relatively longer bones should be advantageous for these taxa to better navigate climbing environments, whereas a longer deltoid ridge provides a larger in‐lever for the acromiodeltoid, a major flexor of the shoulder. This longer in‐lever should augment the acromiodeltoid's leverage as it contracts and allow this muscle to better function in flexing the shoulder and retracting the humerus. These specializations for climbing agree with specializations previously reported for scansorial rodents (Samuels & Van Valkenburgh, 2008), carnivorans as a whole (Samuels et al., 2013; Van Valkenburgh, 1987), musteloids (Fabre et al., 2013b), and mustelids (Holmes, 1980; Schutz & Guralnick, 2007). While scansorial mustelids have been characterized in past studies as not being particularly specialized for climbing compared to carnivorans as a whole (Leach, 1977; Samuels et al., 2013), the results here indicate that the climbing specializations of scansorial mustelids are present to a degree that clearly distinguishes them from fossorial, natatorial, and generalized mustelids.

**Table 2:** Forelimb functional anatomical traits and their loadings along PC axes 1 and 2

Trait	PC1	PC2
Scapula		
Length	−0.041	0.153
Humerus		
Length	0.306	0.006
AP diameter	−0.302	0.164
ML diameter	−0.047	0.184
Deltoid ridge length	0.438	0.112
Epicondylar breadth	−0.150	−0.100
Radius		
Length	0.410	0.085
AP diameter	−0.278	−0.568
ML diameter	−0.060	0.387
Ulna		
Length	0.273	0.040
AP diameter	−0.166	0.082
ML diameter	0.022	−0.368
Olecranon process length	−0.273	−0.162
Metacarpal III		
Length	0.413	−0.494

Anteroposterior (AP) and mediolateral (ML) diameters were measured at midshaft.

The remaining locomotor specializations largely cluster together in phenotypic space, with natatorial and fossorial mustelids occupying somewhat distinct regions and tending toward relatively more robust long bones and a relatively longer olecranon process (Figure 4: PC2; Figure 6). More robust long bones are likely for bones to withstand the mechanical strains incurred in functioning in dense media such as water while swimming or earth while digging. Moreover, a longer olecranon process grants a longer in‐lever to the triceps muscle group to allow for stronger elbow extension and also permits a greater area of attachment for the ulnar head of the flexor carpi ulnaris. Stronger extension and flexion of, respectively, the elbow and wrist should bolster the muscular force generated in drag‐based swimming and scratch digging. My finding of relatively greater long bone robustness and relatively longer olecranon process corroborates previous findings for rodents (Samuels & Van Valkenburgh, 2008), carnivorans as a whole (Samuels et al., 2013; Van Valkenburgh, 1987), musteloids (Fabre et al., 2013), and mustelids (Botton‐Divet, Cornette, Fabre, Herrel, & Houssaye, 2016; Holmes, 1980; Rose et al., 2014; Schutz & Guralnick, 2007). Notably, the overlapping taxa between fossorial and natatorial mustelids are semi‐aquatic mink (*Mustela luterola* and *Neovison vison*) and fossorial taxa associated with later divergences within Mustelidae (*Melogale* sp., *Ictonyx striatus*, and *Poecilogale albinucha*). More aquatic otters appear to inhabit a more distinct region of phenotypic space associated with a relatively longer metacarpal III. A relatively longer metacarpal III should be associated with a larger manus, which would aid swimming. In contrast, some fossorial taxa (e.g., Meles, Taxidea) also possess a relatively long deltoid ridge (Fig. 6), which would enable stronger shoulder flexion while digging. Generalized mustelids overlap with both fossorial and natatorial mustelids.

Along PC2, there is considerable overlap among locomotor specializations in mustelids. This axis in part represents differences in orientation of the cross‐sectional shape of the radius, with its anteroposterior and mediolateral diameters having the lowest and highest loadings for this axis, and a trade‐off between a mediolaterally robust humerus or mediolaterally robust ulna (Table 2; Figure 4). The large overlap among mustelids along PC2 likely reflects among taxa subtle variations among these traits. However, the length of metacarpal III also has a high loading along PC2. Notably, the sea otter (*Enhydra lutris*) has an outlier position along this axis, likely due to the reduced size of its manus relative to other mustelids.

The deltoid ridge is a trait that strongly influences the morphological disparity of mustelids on PC1 (Table 2). However, it should be noted that the morphology of the deltoid ridge varies markedly in Mustelidae. In some taxa, especially badgers and weasels, the deltoid ridge bears a strongly developed deltoid tuberosity. However, many taxa, in particular martens, lack this distinct tuberosity, having instead only a strongly developed deltoid ridge (Fig. 6: compare Meles meles vs. Martes flavigula).

### 4.2 Trait diversification

The best fitting models for both PCs were OU models without a priori defined selective regimes (Table 3). The best fitting model for PC1 locates a single shift in selective regime along the branch leading to Guloninae—the lineage encompassing scansorial mustelids as well as the wolverine (*Gulo gulo*) (Figure 5). In contrast, fossorial and natatorial mustelids, while seeming to occupy somewhat distinct regions of phenotypic space, fall under one selective regime along with generalized mustelids. These results suggest that evolution of the forelimb skeleton under a single phenotypic optimum can facilitate multiple locomotor specializations among mustelids. More specifically, the traits of relatively short and robust long bones and an elongate olecranon process are compatible with specializations for digging, swimming, and terrestrial locomotion. In contrast, the osteological traits suited to scansoriality—relatively elongate and gracile bones, a reduced olecranon process—require a shift to a new selective regime. It is worth noting, however, that the second best fitting model for PC1 is the multi‐optima OU, which may reflect some of the morphological differences between fossorial and natatorial mustelids discussed above. However, overlap in the confidence intervals for phenotypic optima for fossorial, natatorial, and generalized mustelids (Table S2) indicate that these optima cannot be robustly differentiated and further suggest the unconstrained model is the best fitting model for PC1.

**Table 3:** AICc scores for fitted models

Model	L	Rel.L	AICc	ΔAIC	Weight
PC1					
**Unconstrained OU (2)**	**61.9**	**1.00**	**−114.6**	**0.0**	**55.1**
Single‐rate BM	52.0	0.0006	−99.7	14.9	0.0003
Multirate BM	58.2	<0.0001	−95.9	18.8	<0.0001
Single‐optimum OU	52.3	0.0002	−97.9	16.7	0.0001
Multi‐optima OU	64.4	0.819	−114.2	0.41	44.8
Early burst	52.3	0.0002	−97.9	16.7	0.0001
White noise	32.5	<0.0001	−60.7	53.9	<0.0001
PC2					
**Unconstrained OU (3)**	**68.3**	**1.00**	**−124.8**	**0.0**	**99.9**
Single‐rate BM	47.8	<0.0001	−91.2	33.6	<0.0001
Multirate BM	53.9	<0.0001	−96.1	28.7	<0.0001
Single‐optimum OU	52.3	<0.0001	−97.9	26.9	<0.0001
Multi‐optima OU	53.2	<0.0001	−91.9	32.9	<0.0001
Early burst	52.3	<0.0001	−97.9	26.9	<0.0001
White noise	50.7	<0.0001	−97.2	27.6	<0.0001

“Unconstrained” refers to an Ornstein–Uhlenbeck (OU) model without a selective regime defined a priori. Multi‐optima OU and multirate Brownian motion (BM) models have, respectively, four optima and four rates based upon locomotor specializations of fossorial, natatorial, scansorial, and generalized habits. “Early burst” refers to a model of trait evolution in which the rate of evolution is initially high but declines toward the present, whereas “white noise” refers to a model with a complete lack of phylogenetic influence. “L” and “Rel.L” denote log likelihood and relatively likelihood, respectively. “AICc,” “ΔAIC,” and “Weight,” respectively, denote the Akaike information criterion for small samples, the difference in AICc score between a given model and the best fitting model, and the Akaike weight. The best fitting model is highlighted in bold. Numbers in parentheses for unconstrained OU models indicate the number of selective regimes recovered by the model.

The OU model best fitting PC2 had three phenotypic optima (Figure 3). However, two of these optima are for single taxa—the sea otter (*Enhydra lutris*) and the steppe polecat (*Mustela eversmanii*)—with all remaining sampled mustelids falling under a single optimum. Notably, *E. lutris* and *M. eversmanii* have the highest and lowest scores along PC2. The reduced manus of the sea otter, its extensive use in prey manipulation and tool use, and its complete lack of a role during swimming (Kenyon, 1969) likely entail this taxon's shift to its own phenotypic optimum. In contrast, the optimum associated with *M. eversmanii* might be an artifact of this taxon having the lowest score along PC2, as the posterior probability of its associated shift in phenotypic optima is quite low (0.25), and this taxon clusters strongly with other mustelids (excluding *E. lutris*). Given the strong overlap in PC2 scores among remaining mustelids, it does not seem surprising that a single phenotypic optimum characterizes all sampled taxa apart from *E. lutris* and *M. eversmanii*.

### 4.3 α‐values and likelihood ratio simulations

**Figure 7:**
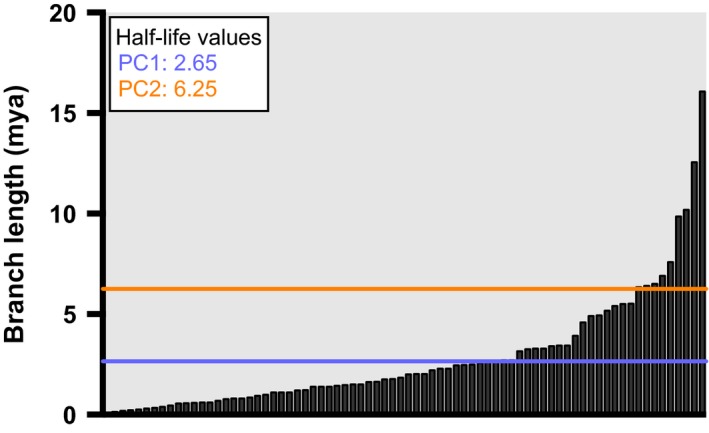
Branch lengths from the phylogeny used in this study. The branch lengths consist of all 76 branches comprising the phylogeny, ordered shortest to longest. The values of phylogenetic half‐life for PC1 and PC2 are superimposed with horizontal lines

Key to interpretation of OU models is an examination of α (Cooper et al., 2016), a parameter often interpreted as the strength of selection, the rate of adaptation, or a “rubber band” parameter (Beaulieu et al., 2012; Butler & King, 2004; Cooper et al., 2016; Uyeda & Harmon, 2014). A more straightforward means to interpret α is to transform it into the phylogenetic half‐life: *t*_1/2_ = ln(2)/α. When *t*_1/2_ is small relative to the branches of the phylogeny, it indicates that the rate of evolution toward the trait optimum is fast and that the influence of past of trait values is weak (Cooper et al., 2016; Hansen, 1997). In contrast, when *t*_1/2_ is large relative to the height of a phylogeny, then it indicates that attraction to phenotypic optima is weak. For PC1, *t*_1/2_ = 2.65 million years. Comparing this value to the mustelid phylogeny's branch lengths (Figure 7) suggests a strong acting OU process that is still influenced by past trait values, especially as some branch lengths are much shorter than *t*_1/2_. The phylogenetic half‐life of PC2 is 6.25 million years. Thus, it would seem that PC2 is under a greater phylogenetic influence than PC1.

Notably, the observed likelihood ratio falls outside of the confidence limits of likelihood ratios generated from data simulated from single‐rate Brownian motion (the null model) and the best fitting OU model. Although the best fitting OU model for PC1 makes biological sense with regard to limb functional anatomy, the results of the likelihood ratio test strongly suggest that the phylogeny used in the present study likely has too few taxa to robustly test OU models (Boettiger et al., 2012; Cooper et al., 2016). Thus, the best fitting model of selective regimes associated with scansorial and nonscansorial mustelids should be regarded with some caution. Likewise for PC2, the observed likelihood ratio falls between the confidence limits obtained from data generated by Brownian motion and the best fitting OU model, indicating that the best fitting model for the morphology represented by this axis should also be treated with caution. One possible means to improve to possibly improve the model accuracy and adequacy of fit is the inclusion of fossil mustelids in future study (Cooper et al., 2016; Marshall, 2017; Schnitzler, Theis, Polly, & Eronen, 2017).

While the location of regime shifts in the differing OU models makes sense in light of functional anatomy and observed locomotor specializations of extant mustelids, it does not necessarily guarantee that the best fitting OU models are the best descriptors of the data. While it was recently recommended for OU models to be fit to datasets of *N* ≥ 200 taxa (Cooper et al., 2016), datasets of functional anatomy seldom include that many taxa. Arguably functional anatomical traits are ideal for studies of selective regimes and adaptive diversification, as principles of physics and engineering theory easily allow for a priori predictions of possible selective regimes. Furthermore, functional anatomical traits can allow an organism to physically function in its environment, and the development of novel functional traits can aid expansion into newly opened ecological niches. Hopefully new or revised OU models can be developed that are ideally suited for the smaller datasets more common for functional anatomical data.

## 5 CONCLUSIONS

Skeletal traits associated with limb function can distinguish mustelids specialized for climbing from remaining mustelids and, to a lesser extent, mustelids specialized for digging from those specialized for swimming. Fitting PC scores to models of trait diversification uncovers that adaptive diversification as the best fitting scenario of the evolution of the forelimb skeleton in mustelids, with a selective regime distinguishing climbing specialists from other mustelids. However, the testing the robustness of model fits finds that the phylogeny of sampled mustelids may have too few taxa to accurately model trait diversification. Including fossil mustelids in future studies may provide one means of improving model fitting. These results underscore the need for comparative methods suited for datasets of functional anatomical traits that are often constrained to sample relatively few taxa.

## CONFLICT OF INTEREST

None declared.

## AUTHOR CONTRIBUTION

B.M. Kilbourne involved in data collection and analysis, figure preparation, and manuscript writing.

## DATA ACCESIBLITY

The doi for this study's data is 10.5061/dryad.87pg9.
