# Supplementary material for: Selective regimes and functional anatomy in the mustelid forelimb: Diversification toward specializations for climbing, digging, and swimming
Source: Ecol Evol. 2017 Sep 20;7(21):8852–63. doi: 10.1002/ece3.3407 (PMC5677490; doi:10.1002/ece3.3407)
Supplement: Supplementary file 1 [file ECE3-7-8852-s001.pdf]

Table S1. Model parameters for a single optimum Ornstein-Uhlenbeck model. σ^2 is the rate of evolution, α is the strength of selection, and Θ is the phenotypic optimum. 95% confidence limits for Θ were obtained by multiplying the standard error by 1.96, the critical value corresponding to a cumulative probability equal to 0.975 for the t-distribution (Beaulieu et al., 2012). Color of taxon names denote the locomotor habits of sampled taxa as a reference: fossorial (brown), natatorial (blue), scansorial (green), and generalized (black).

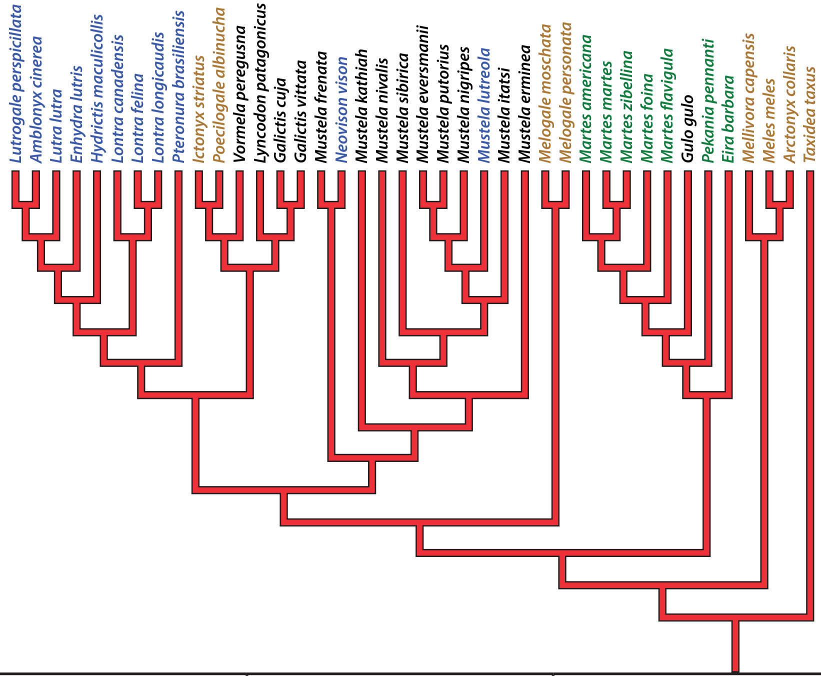

Trait	σ^2	α	Θ
PC1	0.001 ± 0.609	0.025 ± 2.764	-0.003 ± 0.083
PC2	0.003 ± 1.038	0.270 ± 1.159	0.007 ± 0.027

Table S2. Model parameters for a multi-optima Ornstein-Uhlenbeck model. σ^2 is the rate of evolution, α is the strength of selection, and Θ_F, Θ_N, Θ_S, Θ_G and are phenotypic optima for fossorial, natatorial, scansorial, and generalized mustelids. 95% confidence limits for Θ were obtained by multiplying the standard error by 1.96, the critical value corresponding to a cumulative probability equal to 0.975 for the t-distribution (Beaulieu et al., 2012). Color of taxon names denote the locomotor habits of sampled taxa as a reference: fossorial (brown), natatorial (blue), scansorial (green), and generalized (black).

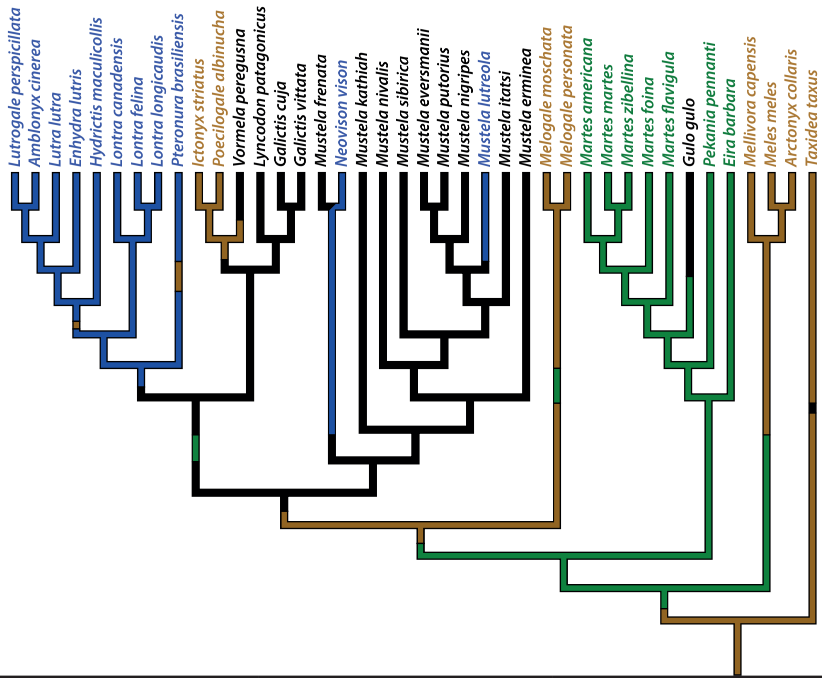

Trait	σ^2	α	Θ_F	Θ_N	Θ_S	Θ_G
PC1	0.573 ± 1.123	0.460 ± 1.134	-0.043 ± 0.038	-0.087 ± 0.038	0.193 ± 0.041	-0.019 ± 0.032
PC2	0.003 ± 1.192	0.311 ± 1.316	0.037 ± 0.043	0.009 ± 0.058	-0.006 ± 0.061	-0.011 ± 0.048

Table S3. Model parameters for a single rate Brownian motion model. σ^2 is the rate of evolution and Θ is the ancestral state at the root of the tree. Color of taxon names denote the locomotor habits of sampled taxa as a reference: fossorial (brown), natatorial (blue), scansorial (green), and generalized (black).

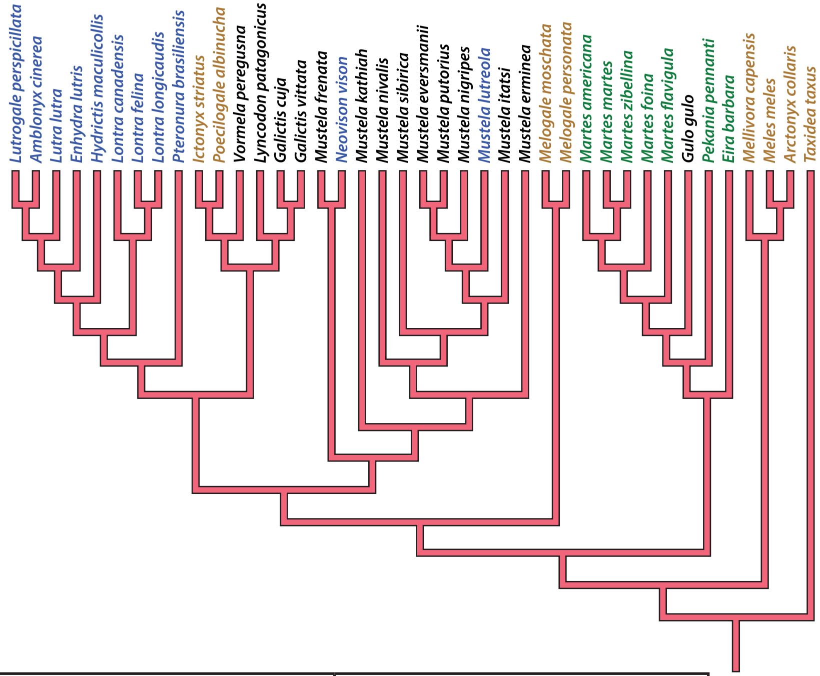

Trait	σ^2	Θ
PC1	0.001 ± 0.438	-0.004 ± 0.102
PC2	0.001 ± 0.438	0.007 ± 0.113

Table S4. Model parameters for a multi-rate Brownian motion model. σ^2_F, σ^2_N, σ^2_S, and σ^2_G are the rates of evolution for fossorial, natatorial, scansorial, and generalized mustelids, respectively. Θ is the ancestral state at the root of the tree. Color of taxon names denote the locomotor habits of sampled taxa as a reference: fossorial (brown), natatorial (blue), scansorial (green), and generalized (black).

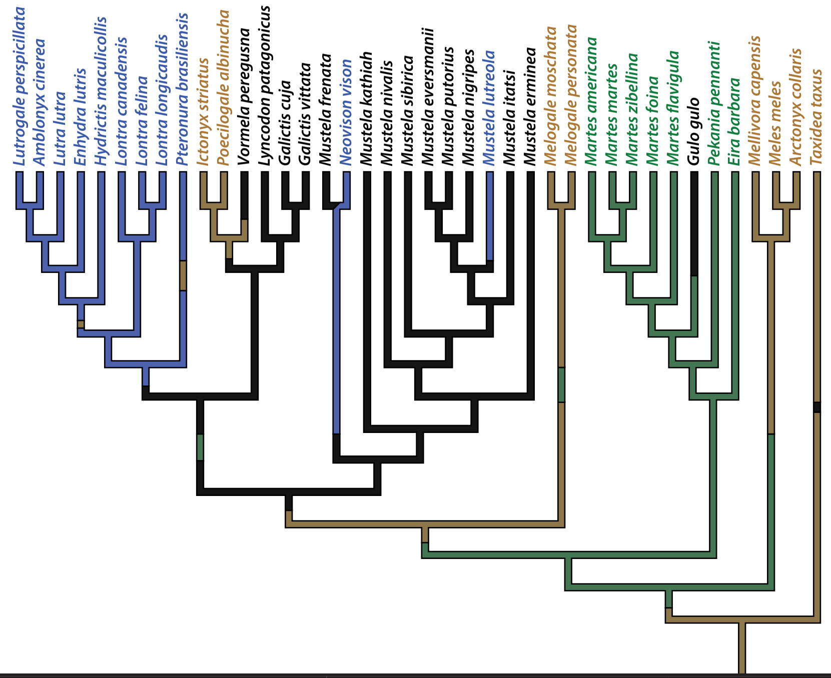

Trait	Θ	σ^2_F	σ^2_N	σ^2_S	σ^2_G
PC1	-0.002 ± 0.098	0.001 ± 1.148	0.001 ± 0.925	0.001 ± 1.311	0.001 ± 0.697
PC2	0.013 ± 0.062	0.000 ± 1.033	0.002 ± 0.877	0.000 ± 1.060	0.001 ± 0.694

Table S5. Model parameters for an Ornstein-Uhlenbeck model for PC1 without an *a priori* selective regime. The phenotypic optima (Θ) are 1, 2, 3, 4, 5, and 6, being numbered as they are first encountered when moving from left to right across the terminal branches of the phylogeny. σ^2 is the rate of evolution, and α is the strength of selection. 95% confidence limits for Θ were obtained by multiplying the standard error by 1.96, the critical value corresponding to a cumulative probability equal to 0.975 for the t-distribution (Beaulieu et al., 2012). Color of taxon names denote the locomotor habits of sampled taxa as a reference: fossorial (brown), natatorial (blue), scansorial (green), and generalized (black).

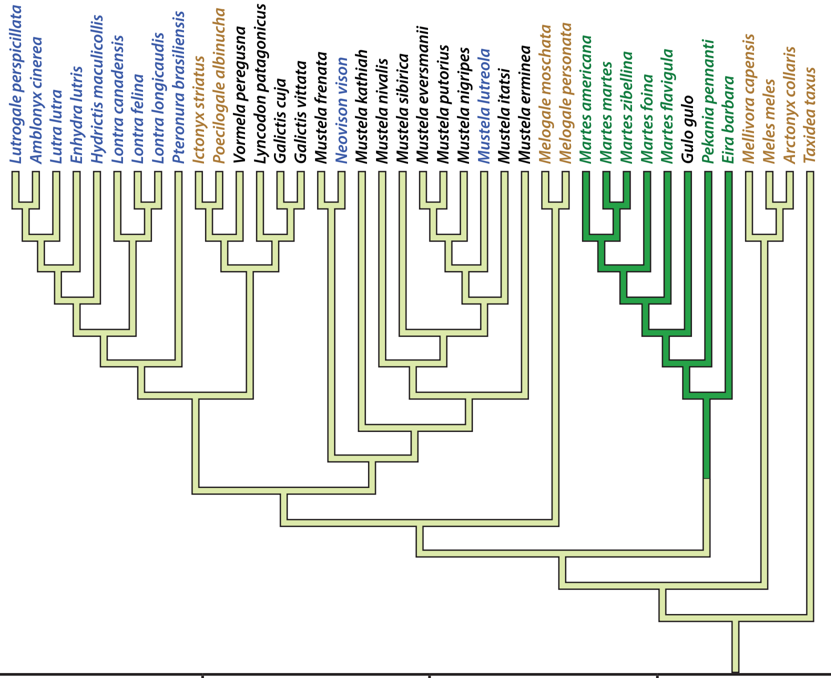

Trait	σ^2	α	Θ_1	Θ_2
PC1	0.002 ± 0.913	0.262 ± 0.996	-0.047 ± 0.025	0.194 ± 0.051

Table S6. Model parameters for an Ornstein-Uhlenbeck model for PC2 without an *a priori* selective regime. The phenotypic optima (Θ) are 1, 2, and 3, being numbered as they are first encountered when moving from left to right across the terminal branches of the phylogeny. σ^2 is the rate of evolution, and α is the strength of selection. 95% confidence limits for Θ were obtained by multiplying the standard error by 1.96, the critical value corresponding to a cumulative probability equal to 0.975 for the t-distribution (Beaulieu et al., 2012). Color of taxon names denote the locomotor habits of sampled taxa as a reference: fossorial (brown), natatorial (blue), scansorial (green), and generalized (black).

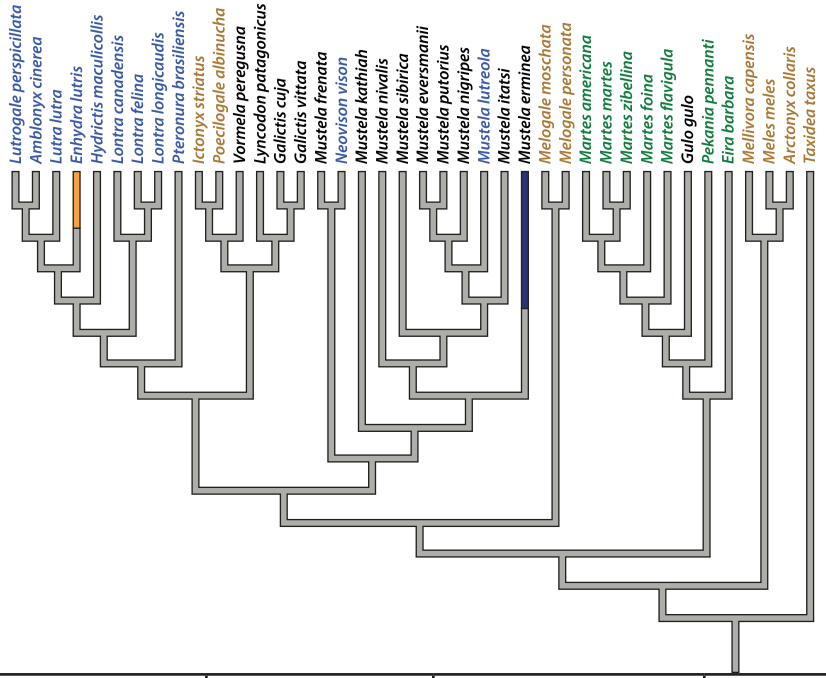

Trait	σ^2	α	Θ_1	Θ_2	Θ_3
PC2	0.001 ± 0.813	0.111 ± 1.285	0.002 ± 0.031	-28.212 ± 14.502	9.099 ± 2.846
